# Urinary Copper Elevation in a Mouse Model of Wilson's Disease Is a Regulated Process to Specifically Decrease the Hepatic Copper Load

**DOI:** 10.1371/journal.pone.0038327

**Published:** 2012-06-22

**Authors:** Lawrence W. Gray, Fangyu Peng, Shannon A. Molloy, Venkata S. Pendyala, Abigael Muchenditsi, Otto Muzik, Jaekwon Lee, Jack H. Kaplan, Svetlana Lutsenko

**Affiliations:** 1 Department of Physiology, Johns Hopkins University, School of Medicine, Baltimore, Maryland, United States of America; 2 Department of Radiology, University of Texas Southwestern Medical Center, Dallas, Texas, United States of America; 3 Department of Biochemistry and Molecular Genetics, University of Illinois at Chicago, Chicago, Illinois, United States of America; 4 Carman and Ann Adams Department of Pediatrics and Department of Radiology, Wayne State University, School of Medicine, Detroit, Michigan, United States of America; 5 Redox Biology Center, Department of Biochemistry, University of Nebraska, Lincoln, Nebraska, United States of America; Auburn University, United States of America

## Abstract

Body copper homeostasis is regulated by the liver, which removes excess copper via bile. In Wilson's disease (WD), this function is disrupted due to inactivation of the copper transporter ATP7B resulting in hepatic copper overload. High urinary copper is a diagnostic feature of WD linked to liver malfunction; the mechanism behind urinary copper elevation is not fully understood. Using Positron Emission Tomography-Computed Tomography (PET-CT) imaging of live *Atp7b^−/−^* mice at different stages of disease, a longitudinal metal analysis, and characterization of copper-binding molecules, we show that urinary copper elevation is a specific regulatory process mediated by distinct molecules. PET-CT and atomic absorption spectroscopy directly demonstrate an age-dependent decrease in the capacity of *Atp7b^−/−^* livers to accumulate copper, concomitant with an increase in urinary copper. This reciprocal relationship is specific for copper, indicating that cell necrosis is not the primary cause for the initial phase of metal elevation in the urine. Instead, the urinary copper increase is associated with the down-regulation of the copper-transporter Ctr1 in the liver and appearance of a 2 kDa Small Copper Carrier, SCC, in the urine. SCC is also elevated in the urine of the liver-specific *Ctr1* ^−/−^ knockouts, which have normal ATP7B function, suggesting that SCC is a normal metabolite carrying copper in the serum. In agreement with this hypothesis, partially purified SCC-Cu competes with free copper for uptake by Ctr1. Thus, hepatic down-regulation of Ctr1 allows switching to an SCC-mediated removal of copper via kidney when liver function is impaired. These results demonstrate that the body regulates copper export through more than one mechanism; better understanding of urinary copper excretion may contribute to an improved diagnosis and monitoring of WD.

## Introduction

Copper is an essential trace element that is required for catalytic activity of key metabolic enzymes involved in oxidative stress response, respiration, blood clotting, and many other physiologic processes. Deficit of copper in the diet is associated with cardiac hypertrophy and neurologic abnormalities, whereas genetically induced copper insufficiency (in Menkes disease or as a result of genetic manipulations in animals) is lethal [1]. Although copper is essential for normal growth and development, excessive copper is toxic. This toxicity is best illustrated by Wilson's disease (WD), a potentially lethal human disease caused by inactivating mutations in the copper transporter ATP7B and massive copper accumulation in the liver [2]. Normally, in humans and other mammals, liver is the main organ that maintains copper homeostasis. In hepatocytes, ATP7B transports excess copper into vesicles for subsequent excretion into the bile [3]. In a healthy organism, this is the primary route of copper removal and little copper is found in the urine.

In WD, the copper excretion pattern changes dramatically. Inactivation of ATP7B impairs copper export into the bile. Copper content of the liver becomes greatly elevated causing, with time, significant pathologic changes in liver morphology and function [2]. Patients with WD also have high urinary copper (208 to 466 µg per day), which serves as an important diagnostic marker for the disease [4], [5]. The origin of high copper in WD urine is not obvious because most of the incoming copper is thought to be trapped in the liver. Disease-induced cell necrosis may result in the release of copper from damaged hepatocytes into the bloodstream. However, experiments directly testing this hypothesis are lacking. Furthermore, elevated copper in the urine is observed in several other conditions (hepatitis, cancer, pregnancy) [6]–[8], when ATP7B function is presumed normal, suggesting existence of a secondary mechanism for copper excretion. A better mechanistic understanding of copper elevation in the urine would facilitate early diagnosis of WD and the treatment monitoring. Consequently, we utilized the *Atp7b^−/−^* mice, an established model for WD, to investigate the basis of urinary copper elevation.

*Atp7b^−/−^* mice, like WD patients, accumulate copper first in the liver and later in other tissues and have a marked liver pathology [9]. Without intervention of chelation therapy, the disease in these animals progresses through three major stages. At Stage I (up to 6–8 weeks after birth), copper accumulates rapidly in the liver and induces changes in cell cycle machinery and lipid metabolism; however no major histological changes are apparent [10]. At Stage II (12–20 weeks), there are numerous metabolic changes, and liver shows clear signs of inflammation, necrosis, and bile ducts proliferation. In animals older than 30 weeks (Stage III), there is a significant recovery of liver morphology and function [11] along with copper sequestration in highly concentrated deposits, appearance of regenerating nodules and continuing bile ducts proliferation [11]. In the present study, we used *Atp7b* ^−/−^ mice to show that the loss of ATP7B-mediated copper export also results in activation of a specific secondary export pathway that diminishes copper load in the liver. This pathway involves time-dependent down-regulation of the copper uptake transporter *Ctr1* in the liver and upregulation of a distinct small copper carrier(s), SCC, in the urine.

## Results

### Copper in the urine of Atp7b^−/−^ mice increases with age but does not directly follow liver pathology

**Figure 1:**
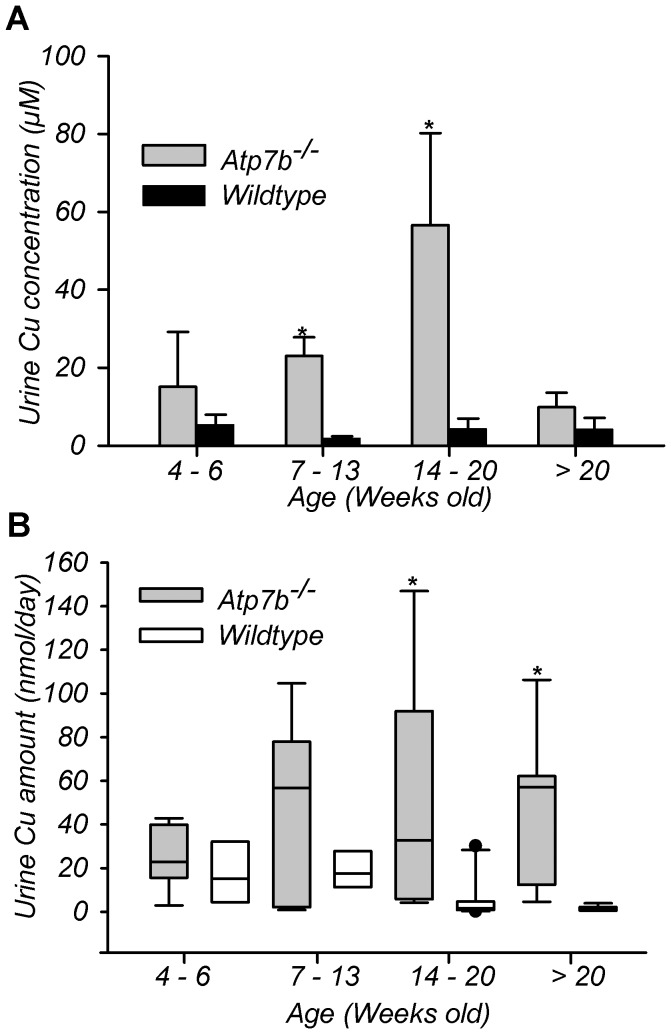
*Atp7b* inactivation induces age-dependent changes in urine copper content. (A) Urinary copper concentration and (B) total amount from wild-type (WT) and *Atp7b* ^−/−^ (KO) mice at different ages. Urine collected over 24 hours and measured by atomic absorption. Data reported as mean concentration ± SD, n = 3 to 13, animals per age group. *P<0.05 versus age-matched wild type. Boxplot middle horizontal bar represents the median; the dotted horizontal line signifies the mean. The while upper and lower box values signify the 75^th^ and 25^th^ percentiles, whiskers represent the 10^th^ and 90^th^ percentiles. The black dots represent outliers (three SD from the mean).

In *Atp7b^−/−^* mice, the liver morphology and function are most impaired at Stage II of the disease (12–20 weeks of age), and both parameters are improved in older animals [11]. Consequently, we tested whether the urinary copper follows liver pathology in *Atp7b^−/−^* mice by measuring copper concentration and total copper output in the urine of animals of various ages. In wild-type mice, urinary copper output and copper concentration were largely unchanged with some decrease in the total copper output observed in animals older than 14 weeks. In contrast, the amount of copper excreted in the urine of *Atp7b^−/−^* mice increased with age (Figure 1). A marked increase in copper concentration was detected between 7 and 20 weeks; a statistically significant change in both concentration and total copper output was most pronounced at 14–20 weeks (Figure 1). This increase coincides with marked pathologic changes in the liver [11]. In animals older than 20 weeks (when copper levels in the liver decreases and liver morphology and function are partially restored) the amount of copper in the urine remained high and the total output was similar to that at 14–20 weeks. Thus, inactivation of *Atp7b* produces age-dependent elevation of copper export through the kidney, which cannot be fully explained by liver necrosis.

### Upregulation of copper export is followed by changes in renal function

**Figure 2:**
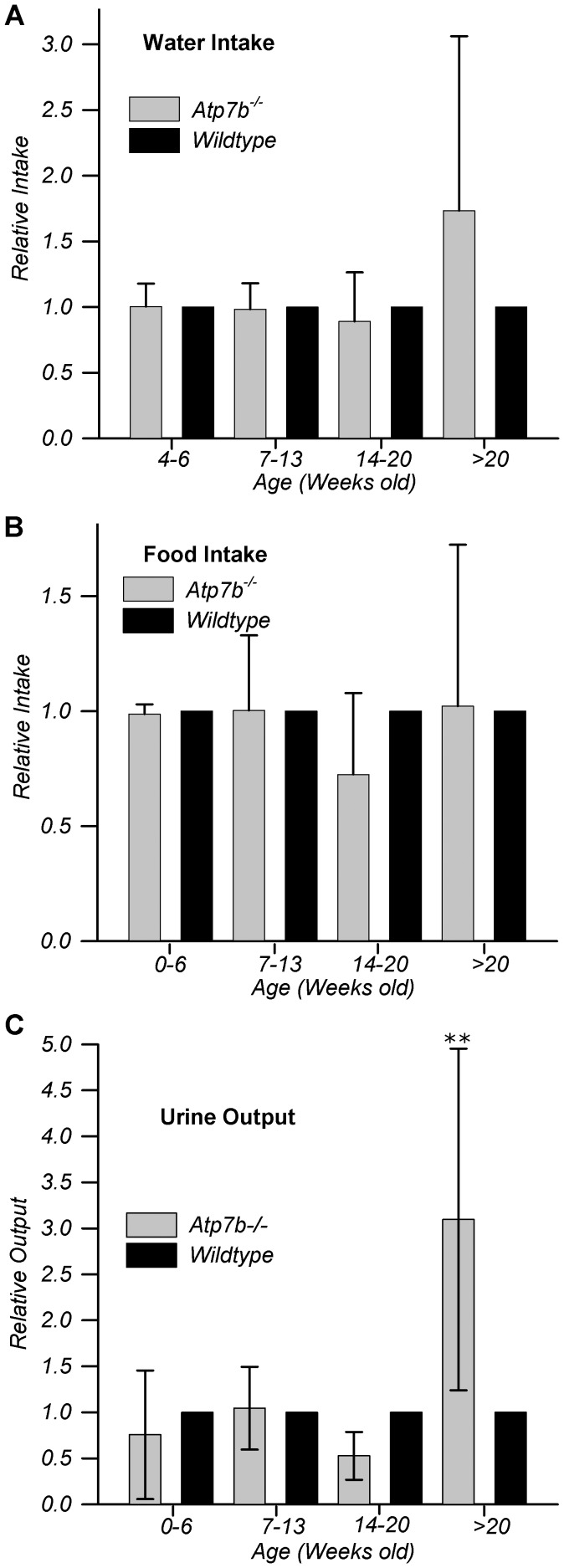
Renal function is altered in *Atp7b* ^−/−^ mice older than 20 weeks. Water intake (A), Food intake (B) and urine volume output (C) relative to age-matched wild-type mice were measured over a 24 hr period. Double asterisks denote p≤0.003. N = 3 to 7 age-matched pairs; data presented as mean ± SD; additional supporting data and details in Figure S1 and information S1.

We also noticed that although total copper output was high in *Atp7b^−/−^* mice older than 20 weeks (Figure 1B), the urinary concentration of copper of these animals was significantly lower compared to younger animals (Figure 1A). To gain a better insight into variations of urinary copper concentration, we measured food and water intake as well as total urine volume (Figure 2). Compared to age-matched controls, food (Figure 2B) and water intake (Figure 2A) did not differ significantly for *Atp7b^−/−^* animals before 20 weeks; however, after 20 weeks both water intake (Figure 2A) and urine volume (Figure 2C) increased dramatically, explaining the decrease in urinary copper concentration at this age. Markedly increased urine volume suggested that renal function was altered in *Atp7b^−/−^* animals older than 20 weeks. This conclusion was confirmed by measuring protein amounts in the urine, which revealed proteinuria in *Atp7b^−/−^* mice older than 20 weeks, but not before this age (Figure S1, additional details in Information S1).

### Longitudinal PET-CT using ^64^CuCl_2_ demonstrates age-dependent changes in copper accumulation by Atp7b^−/−^ liver

**Figure 3:**
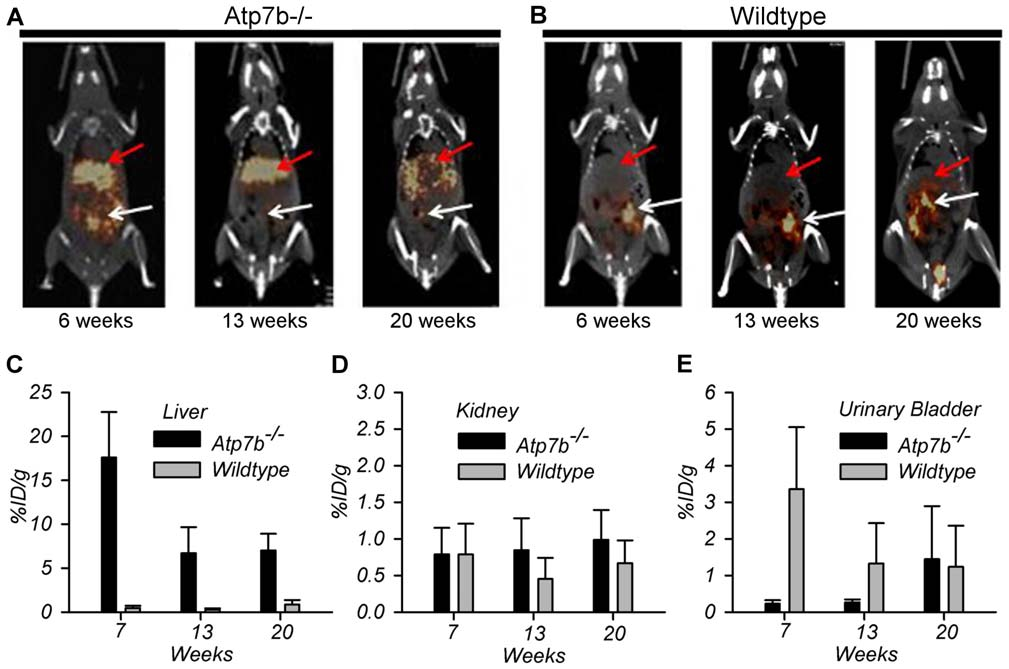
PET-CT analysis of ^64^Cu distribution in live mice at 24 hrs after oral administration of ^64^CuCl_2_. Representative PET-CT images of (A) *Atp7b* ^−/−^ and (B) wild-type mice at 24 hours post oral administration (PO) of ^64^CuCl_2_. Orange-brown color denotes ^64^Cu radioactivity. Red arrows and white arrows identify liver and gastrointestinal tract ^64^Cu radioactivity, respectively. (C) Hepatic ^64^Cu radioactivity in *Atp7b^−/−^* and wild-type mice at 24 hrs PO (p<0.001). Age-dependent ^64^Cu radioactivity in the (D) kidneys (p = 0.38 for group effect) and (E) urinary bladder of wild-type and *Atp7b* ^−/−^ mice at 24 hr PO. %ID/g, percentage of administration dose per gram. Data presented as mean ± SD. N = 5, number of mice with same age and genotype. Additional data and details in Figure S2 and Information S1.

The age-dependent increase of urinary copper suggested that copper uptake and/or excretion by organs were altered in *Atp7b^−/−^* animals in an age-dependent manner. To test this hypothesis we conducted a longitudinal PET imaging of copper distribution in live *Atp7b^−/−^* mice, who received orally administered ^64^CuCl_2_ at 7–8, 13, and 20 weeks of age. We first compared copper accumulation in the liver at early (2 hours post administration) and late (24 hours post administration) phases (Figure S2 and Figure 3, respectively). At 2 hours, there was no measurable difference between ^64^Cu radioactivity in the wild-type and *Atp7b^−/−^* livers (Figure S2 A, B). However, at 24 hours post oral administration of ^64^CuCl_2_, hepatic ^64^Cu radioactivity was noticeably higher in the *Atp7b^−/−^* mice compared to wild-type at all three ages (Figure 3A and B). Further quantitative analysis of PET data revealed a significantly higher accumulation/retention of copper by the *Atp7b* ^−/−^ liver in younger animals (7–8 weeks) compared to older animals (Figure 3C), p<0.001. No significant differences in hepatic copper accumulation were seen in wild-type animals of different ages (p>0.5).

### Age-dependent changes in copper levels in Atp7b^−/−^ kidneys are inverse to those in the liver and less pronounced

The apparent decrease in liver's ability to accumulate copper at 13–20 weeks and a concomitant increase of urinary copper pointed to the increasing involvement of kidneys in the export of copper from the body. To better understand the role of kidneys in copper processing, we performed quantitative PET analysis for both the kidneys and the bladder. We were able to measure large mean differences even though animal to animal-to-animal variation precluded significance. At 2 hours post ^64^Cu administration, an average ^64^Cu radioactivity in the *Atp7b^−/−^* kidneys was consistently lower compared to wild-type at all time-points (Figure S2). At 24 hours, radioactive copper in *Atp7b^−/−^* kidneys at 7–8 weeks was comparable to that of wild-types, but at 13 and 20 weeks copper levels showed a time-dependent increase and trended toward higher values than in the age matched wild-types (Figure 3D). Background ^64^Cu radioactivity in the intestines made it difficult to compare actual values; nevertheless, the overall trend of increasing ^64^Cu in the bladder of *Atp7b^−/−^* mice with age was evident and consistent with the atomic absorption (AA) measurements (Figure 3E). No such increase was observed in the bladder of wild-type mice, again supporting the AA data. It should be noted that although with increasing age, less ^64^Cu is found in the *Atp7b^−/−^* liver and more ^64^Cu radioactivity is detected in the *Atp7b^−/−^* kidneys, renal copper levels remain considerably lower compared to the liver pointing to tissue-specific differences in the mechanisms of maintaining copper balance (see discussion).

### Selective and non-selective phases in urinary copper elevation

**Figure 4:**
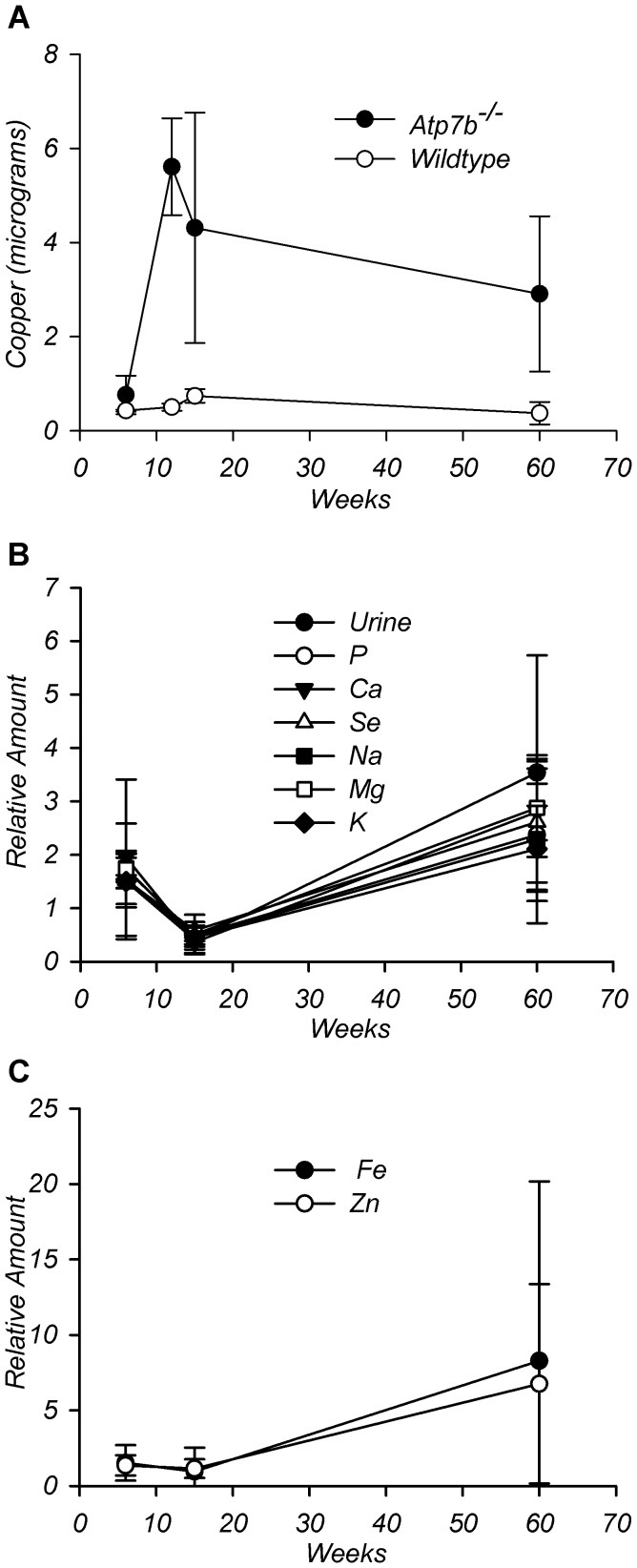
Elemental content of wild-type and *Atp7b* ^−/−^ urine during disease progression. ICP-MS analysis of urine collected over 24 hours from age-matched wild-type and *Atp7b* ^−/−^ mice. (A) Comparison of WT and KO copper amounts at different ages. (B) Urine amounts of Na, Mg, P, K, Ca, Se, and urine volume (relative to wild-type) at different ages. (C) Amounts of Fe and Zn at different ages (relative to wild type). N = 2 to 4 age-match WT/KO pairs for each time point. Data presented as mean ± SD, **P≤0.003. The data points (from left to right) in Panel A represent 6 weeks, 12 weeks, 15 to 20 weeks, and 60 to 65 weeks. Panels B and C data points (from left to right) represent 6 to 12 weeks, 15 to 20 weeks, and 60 to 65 weeks. Correlation coefficients found in Table S1.

Our studies revealed two phases in elevation of urinary copper excretion: an initial phase prior to 20 weeks, when urine volume (and presumably kidney function) were normal, and a later phase (after 20 weeks) when urine volume was markedly increased. To understand the mechanism behind initial copper elevation, we focused on 13–20 weeks animals. We reasoned that elevated urinary copper along with diminished hepatic retention could be due to nonspecific leakage of copper from necrotic *Atp7b^−/−^* hepatocytes since necrosis is observed in the *Atp7b^−/−^* liver [11]. Alternatively, a lower retention of copper in the liver and increase in the urine could be due to specific transport processes such as reduced copper uptake or facilitated export from the liver into circulation. To discriminate between these scenarios, we measured content of several metals in the urine (Figure 4). Specific excretion was expected to produce a selective increase in copper concentration, whereas a mechanism in which hepatic cell death is the primary pathway would likely change levels of several metals [12], [13]. ICP-MS analysis confirmed that Cu levels were high in *Atp7b^−/−^* urine, compared with wild-type and increased with age (Figure 4A). In contrast, the concentration of all other elements did not change significantly between 6 and 20 weeks (Figure 4B). Fluctuations in the total output for all elements but copper showed strong correlation with the changes in urine volume (Table S1, additional details in Information S1), whereas copper was elevated independently of urine volume. Thus, the primary cause of high copper in the urine prior to 20 weeks is a specific change in the inter-organ copper fluxes, consistent with the PET data. In contrast, at 65 weeks of age, all metals showed high levels, especially iron and zinc (Figure 4C). This non-selective change in the urinary elemental content was followed by a marked increase in urine volume and was not explored further.

### Copper in the urine is bound to a Small Copper Carrier (SCC)

**Figure 5:**
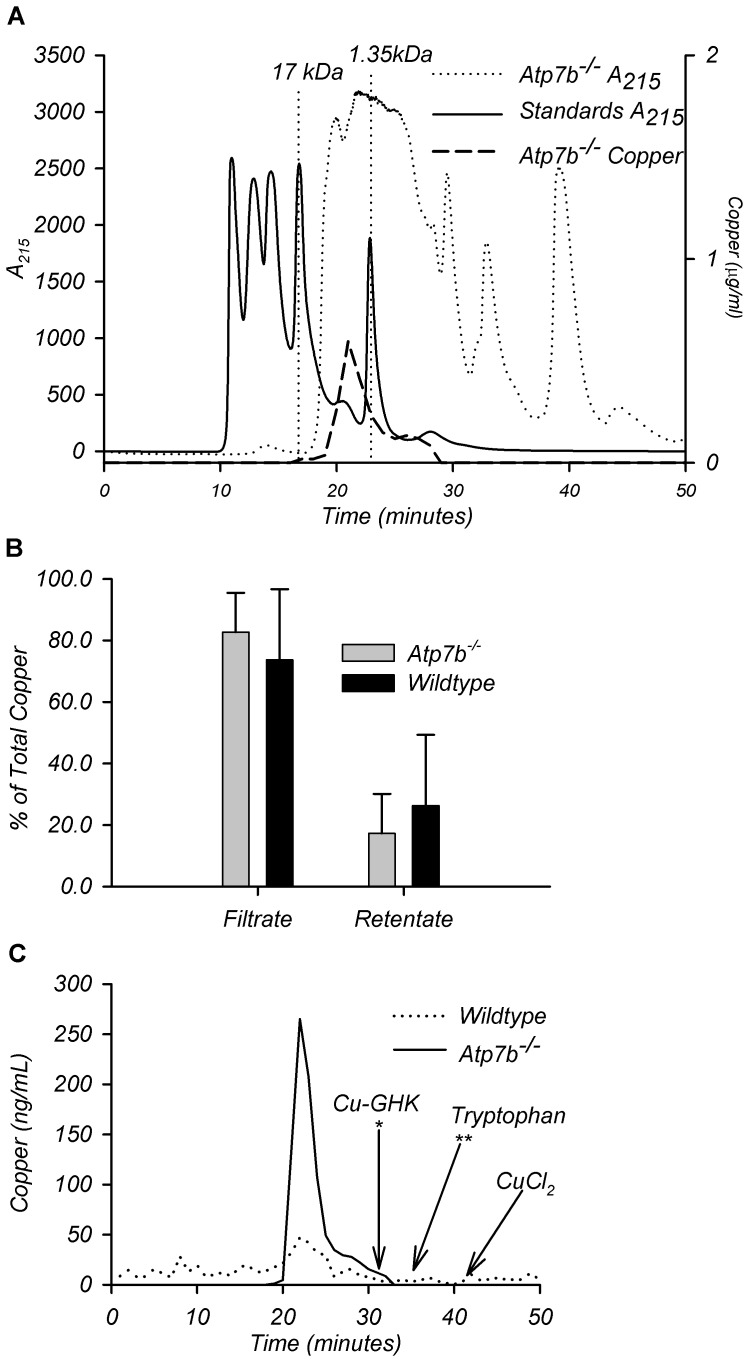
Characterization of urine copper component(s). (A) Representative graph of gel filtration analysis on 20 µl of *Atp7b* ^−/−^ urine along with copper levels in resulting fractions. (B) % of total copper found in the filtrate or retentate of wild-type and *Atp7b* ^−/−^ urine after application of whole urine to a filter with 3 kDa cutoff. Ages varied from 6 to 65 weeks. Data presented as % of total copper ± SD, n = 21 age-matched wild-type/*Atp7b* ^−/−^ pairs. (C) Representative graph of copper profile from gel filtration of 3 kDa filtrate from wild-type and *Atp7b* ^−/−^ urine. Arrows point to the elution time of indicated compounds.

The increase in copper but no other metals raised the question about the molecular form in which copper is exported into the urine. To address this issue, we fractionated urinary copper. Filtering urine through a 3 kDa cut-off filter demonstrated that more than 74% of copper in wild-type and 83% of copper in the urine of *Atp7b* ^−/−^ mice was associated with a low molecular weight fraction. (i.e. found in the flow-through, Figure 5B). High-resolution size-exclusion chromatography of the 3 kDa cut-off ultra-filtrate identified urinary copper in a fraction containing molecules with an apparent molecular weight of 2 kDa (Figure 5A). Subsequent analysis by fractionating CuCl_2_ (to find elution time for free copper), tryptophan (to determine elution time for free amino-acid), and glycyl-L-histidyl-L-lysine copper-binding peptide, GHK (to find elution time for very short peptides) demonstrated that Cu is neither free ion nor bound to free amino-acids, since it elutes earlier, i.e. as a larger molecule (Figure 5C). We hypothesize that the majority of urinary copper binds to a specific Small Copper Carrier(s), which we named SCC.

### CTR1 protein levels show specific age-dependent decrease in the Atp7b^−/−^ liver, whereas ATP7A is unchanged

**Figure 6:**
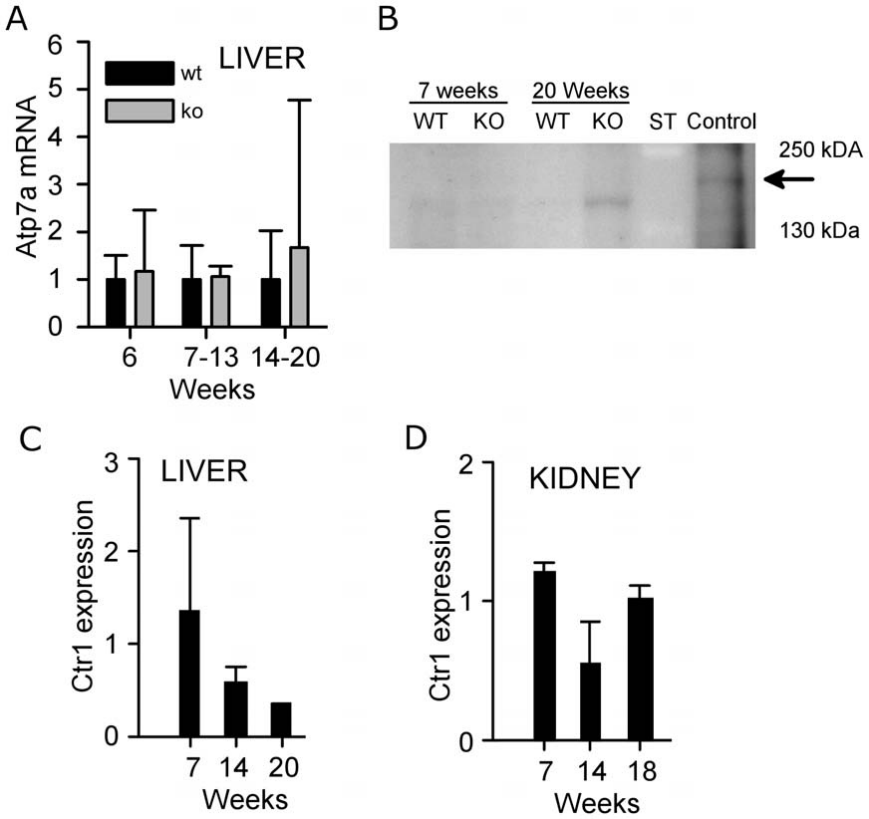
Ctr1 and Atp7A levels in the liver and kidney of Atp7b^−/−^ and wild-type mice. (A) Real-time PCR analysis of Atp7A mRNA levels in the liver of wild-type and Atp7b^−/−^ mice at different ages. Data presented as mean ± SD, n = 3 to 4 different samples of each genotype and age. (B) Representative Western blot illustrating ATP7A protein expression in the livers of wild-type and *Atp7b* ^−/−^ mice at 7 and 20 weeks. Twenty weeks old *Atp7b* ^−/−^ kidneys were used as a positive control for ATP7A protein expression. “ST” denotes molecular weight standards. Black arrow points to ATP7A band. Weak bands in the liver samples are due to non-specific staining (C, D) Quantitation of CTR1 protein levels by Western blot analysis and densitometry (relative to wild type) in liver and kidneys of wild-type and *Atp7b* ^−/−^ mice at different ages. Band intensity in each sample was normalized to a β-actin loading control. Data presented as mean ± SD. n = 2 for each genotype per age group.

The predominant appearance of copper in one peak indicated that copper was bound to a single molecule or a limited number of molecules of a similar size, and implied the involvement of a specific transporter in this process. ATP7A is a copper transporter that can be up-regulated in the liver [14] and thus facilitate copper efflux. Consequently we examined *Atp7a* expression in the *Atp7b^−/−^* liver by real-time PCR and western blotting (Figure 6A,B) when urinary copper levels were near their peak. We found neither significant change in relative mRNA expression nor detectable ATP7A protein in the *Atp7b* ^−/−^ livers. (Figure 6A,B)

We have previously found that the mRNA levels for the copper transporter *Ctr1* are decreased in *Atp7b* ^−/−^ livers [15]. CTR1 is primarily responsible for the high-affinity copper uptake by various tissues, including liver [16]. If the decreased amount of *Ctr1* mRNA is associated with lower protein levels, this would result in a lower copper uptake, explaining (at least in part) the PET data. Consequently, we evaluated the CTR1 protein levels in the wild-type and *Atp7b* ^−/−^ livers at 6, 13, and 20 weeks by Western blot (Figure 6C). When compared to the age-matched wild-types, the *Atp7b* ^−/−^ livers showed age-dependent decrease in CTR1 protein expression. These changes were specific for the liver, since similar analysis of kidney samples showed no significant changes in CTR1 protein compared to those of the wild-type controls (Figure 6D).

### Liver-specific down-regulation of CTR1 is associated with the elevation of copper-bound SCC in the urine

**Figure 7:**
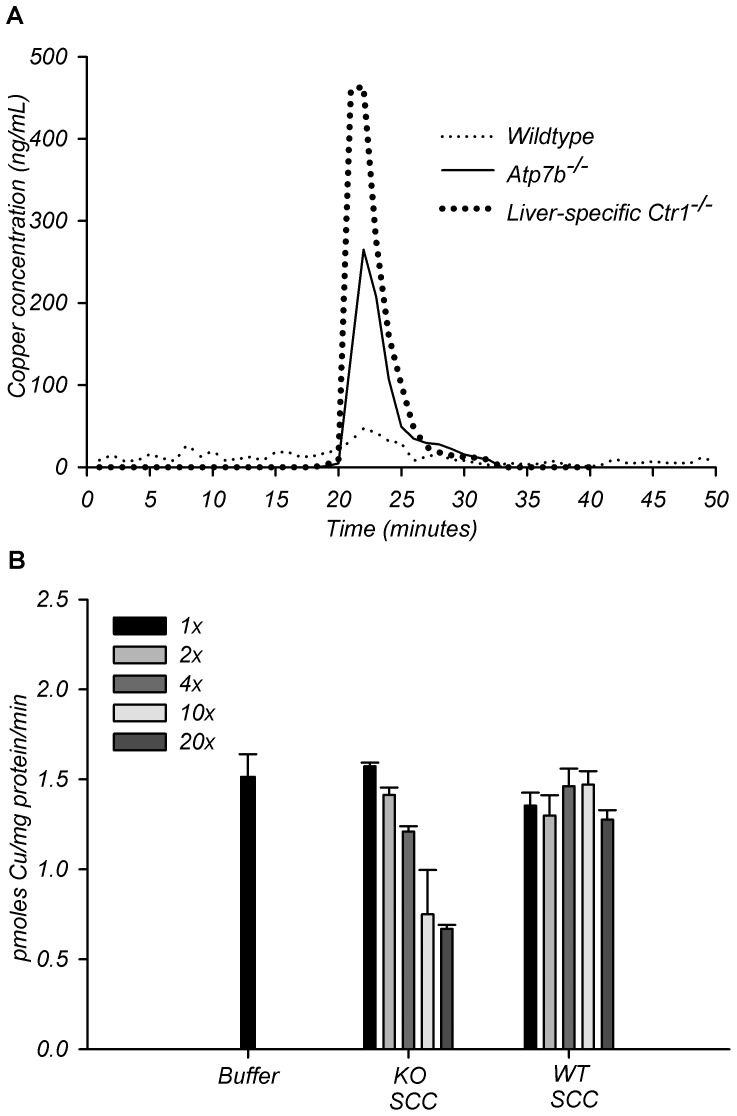
Functional interaction of SCC and CTR1. (A) Copper concentrations in fractions obtained from gel filtration of wild-type, *Atp7b* ^−/−^, and liver-specific *Ctr1* ^−/−^ urine. (B) 64Cu uptake assay by HEK293 cells stably overexpressing hCtr1 in the presence of increasing concentrations of gel filtration purified SCC from urine of *Atp7b* ^−/−^ and wild-type mice. Radioactive copper-64 was kept constant at 0.25 µM. Sodium phosphate buffer pH 7.4 was used as the buffer control. 1× is equal to a copper concentration of 0.25 µM for *Atp7b^−/−^* SCC and 2× to 20× are multiples of the original 1× concentration. The volumes used for the assays were set to achieve the desired 1× to 20× concentrations specifically for *Atp7b^−/−^* SCC. The wild-type SCC 1× to 20× assays are done with the same volumes of SCC utilized for corresponding *Atp7b^−/−^* SCC assays, thus wild-type concentrations used are low due to inherent lower copper levels in WT SCC. Data presented as mean ± SD, performed in triplicates. See Figure S3.

The apparent correlation between CTR1 down-regulation in the liver and elevation of SCC in the urine suggested that these events might be linked. Specifically, we hypothesized that SCC could be a normal source of exchangeable copper in the serum and that Cu-SCC became elevated in the urine because less CTR1 is available to accept copper from SCC and transport into hepatocytes. To test this hypothesis, we examined the urinary copper components of mice that have normal *Atp7b*, but lack *Ctr1* specifically in the liver. These animals do not have liver disease yet have elevated urinary copper [16]. Gel filtration analysis of urine from the liver –specific *Ctr1* knock-out mice showed a single peak of elevated copper that has the same elution time as SCC from *Atp7b* ^−/−^ mice (Figure 7A).

### SCC competes with radioactive ^64^Cu for uptake via CTR1

To further test our hypothesis that SCC represents a source of exchangeable copper in the serum, we measured total and exchangeable copper in wild-type and *Atp7b* ^−/−^ serum at 20 weeks and attempted to detect SCC in the serum. We observed an increase in a fraction of exchangeable copper in Atp7b^−/−^ serum compared to wild-type (Figure S3, additional details Information S1), however we were unable to directly link it to SCC, presumably due to the association of serum copper with a fraction of high-molecular weight proteins (we found that in the mouse sera, copper-bound small molecular weight complexes, such as copper-bound chelators, do not elute with their apparent molecular weight in gel filtration chromatography but rather associate with higher-molecular weight proteins, data not shown).

Consequently, to examine whether SCC can transfer copper to CTR1 we measured competition between Cu-SCC and free Cu using ^64^Cu uptake assays in HEK293 cells stably over-expressing CTR1. Identical volume from the corresponding elution fraction of the wild-type urine served as a control. ^64^Cu uptake was gradually reduced in the presence of increasing amounts of SCC from KO urine. No such competition was seen using the wild-type fractions (Figure 7B).

## Discussion

Genetic mutations in the copper transporter ATP7B disrupt biliary copper excretion resulting in a massive hepatic copper accumulation and Wilson's disease (WD). Using *Atp7b^−/−^* mice, an animal model for WD, we demonstrate that *Atp7b* inactivation also up-regulates an available but underutilized secondary pathway for copper removal from the body, which produces a clinically well-known phenomenon of high urinary copper in WD patients. *Atp7b^−/−^* mice show an age-dependent increase of urinary copper that does not directly follow known changes in liver pathology. This increase is selective for copper in animals under 20 weeks of age, which strongly suggests that urinary copper excretion is a specific mechanism to protect against hepatic copper overload. A similar increase in urinary copper has been documented in WD patients as they transition from asymptomatic to symptomatic stage of the disease [5].

Older *Atp7b^−/−^* mice show changes of several metal ions and a markedly increased urine volume. This latter change is associated with an increased water intake and proteinuria, suggestive of glomerular damage. The time-dependent kidney damage could be either due to increased amounts of copper being filtered through kidney or, more likely, due to inactivation of renal *Atp7b* and copper misbalance in kidney cells. *Atp7b* is normally expressed in kidneys at relatively high levels [17]–[19] and inactivation of *Atp7b* is likely to alter normal copper homeostasis. It is interesting that despite loss of ATP7B function and gradual accumulation of copper in the kidney, renal copper overload is modest compared to the liver. This is likely due to the presence in kidneys of another copper-transporter, ATP7A, which is likely to facilitate copper release partially compensating for the loss of ATP7B function [20].

We have found that copper in the urine is bound to a previously unknown small carrier, SCC, with an apparent molecular weight of 2 kDa; SCC is larger than free amino-acids or the tripeptide GHK, but smaller than metallothionein. Sensitivity of copper-bound SCC to pH and organic solvents has complicated purification using standard protocols, and we are developing new approaches for SCC identification. Importantly, a similar small copper carrier complex is present at lower levels in wild-type urine, which suggest a role for SCC in normal copper metabolism. One such role could be copper sequestration and excretion via renal filtration when hepatic copper uptake is saturated. However, the SCC's ability to compete with ^64^Cu for uptake via CTR1 strongly suggests that its normal physiological function could also be to deliver copper to cells. The highly similar copper profiles of urine from the *Atp7b* ^−/−^ and liver-specific *Ctr1* ^−/−^ mice further illustrates a functional interaction between SCC and CTR1.

Hepatic copper homeostasis is normally achieved by regulation of copper uptake (CTR1), sequestration (MT), and export (ATP7B). In the absence of ATP7B, MT levels are significantly elevated in WD patients and Atp7b^−/−^ mice [10] yet elevation of MT alone cannot explain why copper is shuttled away from the liver as disease progresses. It was suggested in the literature that WD hepatocytes may release the MT-Cu complex [21], however our data are inconsistent with this hypothesis. ATP7A, which is not ordinarily seen in the adult mouse liver, under certain conditions can be upregulated in the liver [14] and pump copper across the basolateral surface back into the blood. Increased ATP7A levels could readily explain the age-dependent increase in serum copper levels observed in *Atp7b* ^−/−^ mice [11]. In the Atp7b^−/−^ mice, we previously documented elevation of ATP7A in the kidney and brain, where ATP7A is assumed to replace some of the biosynthetic functions of ATP7B [20], [22]. Additionally, specific genetic ablation of *Ctr1* in the heart, results in the elevation of ATP7A in the adult mouse liver [14]. However, in the liver of *Atp7b* ^−/−^ mice we did not find any changes in ATP7A protein or mRNA expression. These results agree with the PET data, showing higher copper retention in *Atp7b^−/−^* liver at all time-points despite time-dependent decrease of copper uptake transporter, *Ctr1*.

Walshe has insightfully suggested that in WD patients copper retention by the liver decreases as disease progresses [14]. Longitudinal PET-CT imaging of live *Atp7b*−/− animals allowed direct measurements of copper accumulation in their liver at different ages and tested this hypothesis. It is apparent that radioactive copper found in the liver 24 hours post injection decreases with age. The major copper entry pathway into the liver is through CTR1. CTR1 is known to be regulated by copper at the level of protein stability [23], trafficking [24]–[26], and transcription [27], [28]. We found in the *Atp7b* ^−/−^ mouse liver, CTR1 protein and mRNA levels decrease in an age-dependent manner. The ablation of Ctr1 specifically in the liver of mice does not induce visible pathology in the liver, but is associated with elevation of urinary copper and no other metals [16]. Since no other metal increases in the urine prior to 20 weeks of age, we have concluded that down-regulation of CTR1 and increased filtration of SCC (rather than hepatocytes death) are primarily responsible for the initial phase of urinary copper elevation.

In is important to emphasize that our results do not exclude some contribution of copper from necrotic cells and may reflect a situation when the disease is relatively mild. In humans, the course of WD varies greatly from mild inflammation to a sudden liver failure. Whether the origin and/or molecular identity of urinary copper are the same in these conditions is unclear. The LPP rats, another model of WD, die invariably from liver failure that was linked to mitochondria destruction [29]. Mitochondria matrix contains a small copper ligand CuL [30], which upon mitochondria rupture and cell necrosis would presumably be released. In *Atp7b^−/−^* mice, the course of disease does not include liver failure and the selective mechanism discussed in present work better accommodates our results. Further characterization of copper-carrying small molecules will greatly contribute to our understanding of hepatic copper homeostasis and may help WD diagnosis and monitoring.

In conclusion, elevated urinary copper in *Atp7b* ^−/−^ mice is the result of a redundant and copper-selective mechanism based upon homeostatic control of CTR1 that is turned on to maintain systemic copper balance when liver copper homeostasis is compromised. The redirection of copper flow from the liver to kidneys in WD is mediated by a small copper carrier SCC, which is distinct from free amino acids, tripeptide GHK, or a full-length metallothionein. Functional interactions between SCC and CTR1 may control copper distribution between tissues.

## Materials and Methods

### Materials

Doubly deionized water (ddH_2_O) was used in preparation of buffers and reagents. All buffers were treated with metal-ion chelating agent, Chelex 100 (Bio-Rad) prior to use then subsequently vacuumed degassed and filtered on a 0.45 µM FHLC filter (Millipore). Gel filtration standards were from Bio-Rad Laboratories.

### Animal husbandry and urine collection

Urine was collected from *Atp7b* knockout (*Atp7b* ^−/−^), *Ctr1* knockout and wild-type (WT) littermate control mice. The generation of these mice was previously described [16], [31]. The animals were housed at the Johns Hopkins University School of Medicine Animal Care Facility according to National Institute of Health guidelines. For urine collection, age matched *Atp7b* ^−/−^ and WT mice of both genders were placed for 48 hours in individual metabolic cages (Tecniplast). Animals were allowed to acclimate to their new environment for the first 24 hours, and then urine was collected for the subsequent 24 hour period. During collection, the mice had free access to food and water. Urine volume as well as water and food intake were measured. The urine was centrifuged at 4,000×g for 5 minutes at 4°C to remove debris. Urine was either used immediately or stored at −80°C. The above experimental protocols were approved by the Institutional Animal Care and Use Committee (IACUC) of JHU. Urine collection from the liver specific Ctr1−/− mice was performed as previously described [16] using protocols approved by the IACUC of University of Nebraska-Lincoln. Subsequent analysis of urine from both animal strains was performed under identical conditions, as described below.

### Tissue preparation and analysis

Tissues were dissected after whole animal perfusion with phosphate-buffered saline (pH 7.4; PBS), frozen in liquid nitrogen, and stored at −80°C until use. Protein extracts were prepared on ice by homogenizing liver and kidney in the lysis buffer (50 mM Hepes (pH 7.4), 0.1% Igepal,150 mM NaCl, 1 mM AEBSF, 250 mM sucrose) containing EDTA-free protease inhibitor cocktail (Roche). The tissue homogenates were centrifuged at 700× g for 15 minutes to remove debris and the supernatant was spun at 22,000× g for 35 minutes to obtain a membrane pellet. The pellet was then dissolved in the Laemmli gel sample buffer. For SDS-PAGE, 1 µg and 25 µg of membrane protein were separated on 7.5% and 4–20% polyacrylamide gels to analyze Atp7A and Ctr1, respectively. Proteins were transferred onto polyvinyldifluoride (PVDF) membrane and probed with rabbit anti-CTR1 (1∶1000) and rabbit anti-ATP7A (1∶500) antibody. Staining was detected by goat anti-rabbit HRP secondary antibody (Santa Cruz Biotechnology, 1∶20,000) and HRP-conjugated anti-mouse β-actin (Sigma,1∶10,000) using West Pico Chemiluminescence (Pierce) according to the manufacturer's instructions. Staining with anti-β-actin antibody was used as a loading control. Band intensity was measured with Image J and quantified after normalization to β-actin.

### Longitudinal PET-CT

The PET imaging experiments were conducted under the protocol approved by the Institutional Animal Care and Use Committee, UT Southwestern Medical Center at Dallas. The mice were administered with ^64^CuCl_2_ [74 kBq (2 µCi)/g body weight] in a volume of 25 µl by oral feeding. The dose of ^64^CuCl_2_ supplied in 0.1 N HCl was diluted in 25 µl of normal saline containing 0.9% sodium chloride. PET of the mice was performed with a Siemens Inveon microPET/CT system, using a protocol published recently [32]. Briefly, the mice were anesthetized using 3% isoflurane at room temperature and placed in spread-supine position on the imaging bed under 2% isoflurane anesthesia for the duration of the imaging. Static whole body imaging was performed at 2 and 24 hours post oral administration of the tracer, respectively, which consisted of two overlapping frames of 15 min duration for each frame. As part of the PET imaging protocol, CT data was acquired (80 kV, 500 µA) for attenuation correction and anatomical localization. PET images were subsequently reconstructed using the 3D OSEM algorithm and scatter as well as attenuation correction. All images were analyzed using the Inveon Research Workplace (IRW) software (Siemens). Following re-slicing of fused PET/CT images into arbitrary views, regions of interest (ROIs) for liver, kidneys, and urinary bladder were drawn to obtain quantitative measurements of the ^64^Cu tracer activity expressed as percentage of administration dose per gram (%ID/g).

### Size-Exclusion Chromatography of urine filtrates

Urine was ultrafiltered on a Microcon YM-3 (MWCO 3000, Bedford MA) at 14,000×g for 200 min at 4°C. One hundred microliters of urine filtrate was loaded onto a size-exclusion column (Phenomenex Bio-Sep S-2000, 7.5×200, 10 µM), equilibrated with 50 mM sodium phosphate buffer, pH 7.5. Samples were fractionated isocratically with the same buffer at a flow rate of 0.5 ml per min, and 0.5 ml fractions were collected over 50 minutes. Absorbance was monitored at 215 and 280 nm. Calibration of size exclusion column was done using molecular weight standards (Bio-Rad, Thyroglobulin 670 KDa; γ-globulin 158 KDa; Ovalbumin 44 KDa; Myoglobin 17 KDa; Vitamin B12 1,350 Da), reduced L-glutathione (307 Da), tryptophan (∼204 Da), Copper Chloride (∼64 Da). All standards were prepared in 50 mM sodium phosphate buffer pH 7.5.

### Atomic Absorption Spectrophotometry

The copper concentration of the urine, filtrate, and in the chromatography fractions was determined using a Shimadzu 6650 graphite furnace atomic absorption spectrophotometer (AA) equipped with an ASC-6100 autosampler. Briefly, samples were diluted with ddH_2_O (1∶10 or 1∶500 for fractionated and unfractionated urine, respectively) to be in the linear absorption range of the calibration curve [1–10 parts per billion (ppb)]. Each sample was measured 2 to 3 times, and the concentration of copper was derived by comparing absorption with a calibration curve. All values are reported as means.

### Inductively coupled plasma mass spectrometry (ICP MS)

Analysis was performed using an Agilent 7700× equipped with an ASX 250 autosampler. The system is operated at a radio frequency power of 1550 W, an argon plasma gas flow rate of 15 L/min, Ar carrier gas flow rate of 1.04 L/min. All elements were measured in kinetic energy discrimination (KED) mode using He gas (4.3 ml/min). Urine samples were diluted into 1% HNO_3_, (trace metal grade, Fisher Scientific). Data were quantified using a 10-point calibration curve (0–5000 ppb (ng/L)) with external standards for Na, Mg, P, K, Ca, Fe, Cu, Zn, in 1% HNO3, (to match the sample matrix). For each sample, data were acquired in triplicates and averaged. An internal standard was used to correct for plasma instabilities and differences in total ion concentrations, triplicate measurements of a 10 ppb Cu solution as well as a blank (containing diluent only) was used as quality control. To ensure maximum recovery of elements from the sample certified NIST standard reference materials (serum (SRM 1598a), and water (SRM 1643e)) were prepared and analyzed by the same method as the samples. Time-points presented in Figure 4 represent different ages of mice plotted as one time point; the “6 week” time-point represents the average of data for 6–12 week old urine, the “15 week” timepoint is the average of 15–20 week samples, and the 60 week timepoint is the average of 60–65 week old urine. Four age-matched wild-type/*Atp7b* ^−/−^ pairs were used to generate data for each of the 6 and 15 timepoints; two age-matched wild-type/*Atp7b* ^−/−^ pairs were used for the 60 week time-point.

### Real-Time PCR

Total RNA was isolated from 100 mg of mouse liver using Trizol Reagent (Invitrogen). RNA quality was assessed by Agilent 2100 Bioanalyzer with all samples having RIN values greater than 8.6. For real-time PCR, cDNA was synthesized with QuantiTect Reverse Transcription Kit (Qiagen) from 1 µg of RNA. The target and control genes were amplified in 20 µL reactions using Applied Biosystems (ABI) Taq-Man Universal PCR Master Mix and Taq-Man gene expression primers on an ABI 7500 Real-time PCR system. RT-PCR data was analyzed using the ΔΔCT method. GAPDH was used for normalization.

#### Copper uptake

HEK293 cells containing tetracycline-regulated N-Terminal FLAG-tagged hCTR1 was previously described [33]. The cells were grown at 37°C and 5% CO_2_ in DMEM (Mediatech) containing 10% FBS (Atlanta Biologicals), 25 mM Hepes and 1% PSF (Invitrogen) with the following selective antibiotics: 10 µg/ml blasticidin and 400 µg/ml hygromycin B. hCTR1 protein expression was induced by the addition of 1 µg/ml of tetracycline to the medium for 48 hours.

One day prior to Cu uptake assay HEK/hCTR1-FLAG tagged cells were seeded onto 12-well tissue culture plates at a density of 0.6×10^6^ cells/well. Prior to the uptake assay the media was removed from the cells and replaced with transport media, DMEM+10%FBS, to which either gel filtration fraction of Atp7b^−/−^ urine was added at 0.25–5 µM Cu or the same volume of the fractionated wild-type urine was added in a final volume of 0.5 ml. Control cells were those to which no urine sample was added. Copper uptake was performed by the addition of at 0.25 µM CuCl_2_ containing ^64^Cu (Washington University, St. Louis, MO) to the cells for 45 minutes at 37°C. Cu uptake was halted by the addition of ice-cold stop buffer (150 mM NaCl, 5 mM KCl, 2.5 mM MgCl_2_, 10 mM EDTA, 25 mM Hepes, pH 7.4) to the cells; they were subsequently washed twice with stop buffer. The cells were then lysed in lysis buffer (0.4% Triton X-100, 5 mM DTT, 20 mM Tris pH 8 and 2 mM EDTA) and an aliquot of the cell lysate was added to EcoLume Scintillation fluid and counted using a Beckman LS 6500 scintillation counter. The protein concentration of the cell lysate was calculated after radioactive decay and the copper uptake per well was calculated as pmoles Cu/mg protein/min and the average of triplicate measurements per treatment were expressed as pmoles Cu/mg protein/min.

### Statistical analysis

Values are expressed as mean ± SD. Statistical significance was evaluated using unpaired t-test and among more than two groups by analysis of one-way ANOVA. Differences were considered significant at p<0.05. PET quantitative values are expressed as mean of %ID/g ± SD. In order to assess significant differences in ^64^Cu tracer activity among liver, kidneys, and urinary bladder between *Atp7b* ^−/−^ and C57BL wild-type mice, a 2× (3) mixed-design analysis was conducted, where the between-subjects factor represents the group (*Atp7b* ^−/−^, C57BL mice) and the within-subjects factor represents the three time-points (7–8, 13, 20 weeks). Analyses were performed separately for the 3 regions of interest (liver, kidneys, urinary bladder). The overall test was subsequently followed-up by post-hoc unpaired t-tests between the two groups for individual time-points. A p value<0.05 was considered to represent statistical significance. Box plot outliers were determined by multiplying the interquartile range by 1.5 and subtracting the obtained value from either the first quartile or third quartile, to determine the lower and upper limits; all measurements beyond these limits are considered outliers.

## Supporting Information

Figure S1 **Related to** **Figure 2** **: Proteinuria is apparent in** ***Atp7b*** **^−/−^ mice older than 20 weeks.** Comparison of protein amounts from urine of Atp7b^−/−^ and wildtype mice at different ages. The solid horizontal bar within in the box represents the median, while upper and lower bars signify the 75^th^ and 25^th^ percentiles, respectively. The dotted horizontal line signifies the mean. The whiskers represent the 10^th^ and 90^th^ percentiles. The black dots represent outliers. ^#^P value = 0.088. See supporting information for experimental details (Information S1).
(TIF)

Figure S2 **Related to** **Figure 3** **.** Representative PET-CT imaging and quantitative analysis of wildtype and Atp7b^−/−^ mice at 2 hours post oral administration of ^64^Cu. Pet-CT images of (A) wildtype and (B) Atp7b−/− mice at indicated ages. Quantitative analysis of radioactivity of (C) liver and (D) kidneys of wildtype and Atp7b^−/−^ mice at indicated ages (p = 0.27 for group effect). Data presented as mean ± SD. n = 5, per genotype. Red arrows indicate the position of the liver and white arrows indicate ^64^Cu radioactivity in the gastrointestinal tract.
(TIF)

Figure S3 **Related to** **Figure 7** **: Exchangeable copper in the serum of wild-type and Atp7b^−/−^ mice.** Data presented as mean ± SD. n = 2 per genotype. See supporting information for experimental details (Information S1).
(TIF)

Table S1 **Related to** **Figure 4** **: Correlation between changes in urine volume and amounts of urinary elements with disease progression.** *Spearman ranked coefficients with significance p<0.05. n = 6. See supporting information for description of statistical analysis (Information S1).
(DOCX)

Information S1 **Supplementary experimental procedures.**
(DOCX)
