# Supplementary figures and images for: Urinary Copper Elevation in a Mouse Model of Wilson's Disease Is a Regulated Process to Specifically Decrease the Hepatic Copper Load
Source: PLoS One. 2012 Jun 22;7(6):e38327. doi: 10.1371/journal.pone.0038327 (PMC3390108; doi:10.1371/journal.pone.0038327)

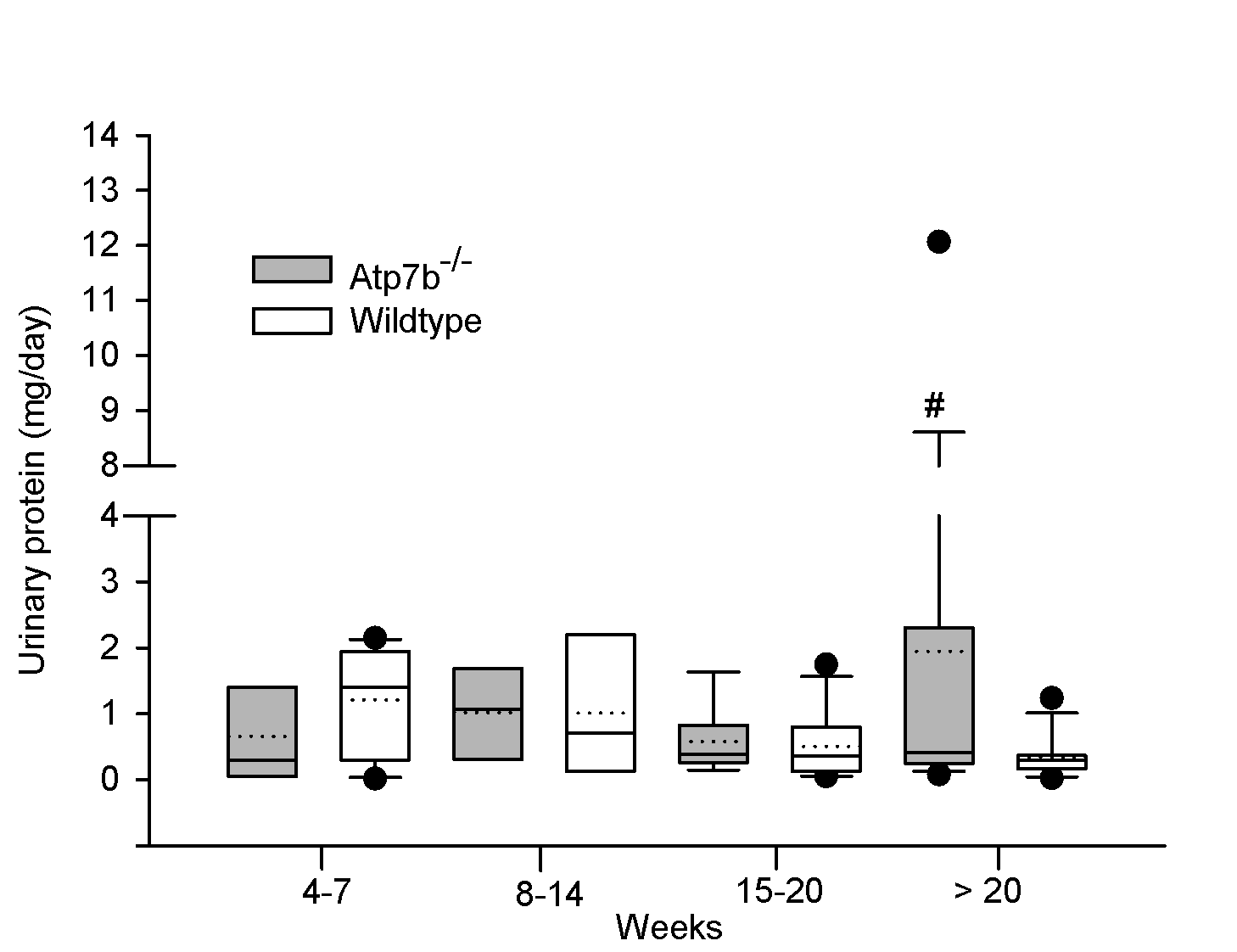

Supplement: Figure S1 — Related to Figure 2 : Proteinuria is apparent in Atp7b −/− mice older than 20 weeks. Comparison of protein amounts from urine of Atp7b−/− and wildtype mice at different ages. The solid horizontal bar within in the box represents the median, while upper and lower bars signify the 75th and 25th percentiles, respectively. The dotted horizontal line signifies the mean. The whiskers represent the 10th and 90th percentiles. The black dots represent outliers. #P value = 0.088. See supporting information for experimental details (Information S1). (TIF) [file pone.0038327.s001.tif]

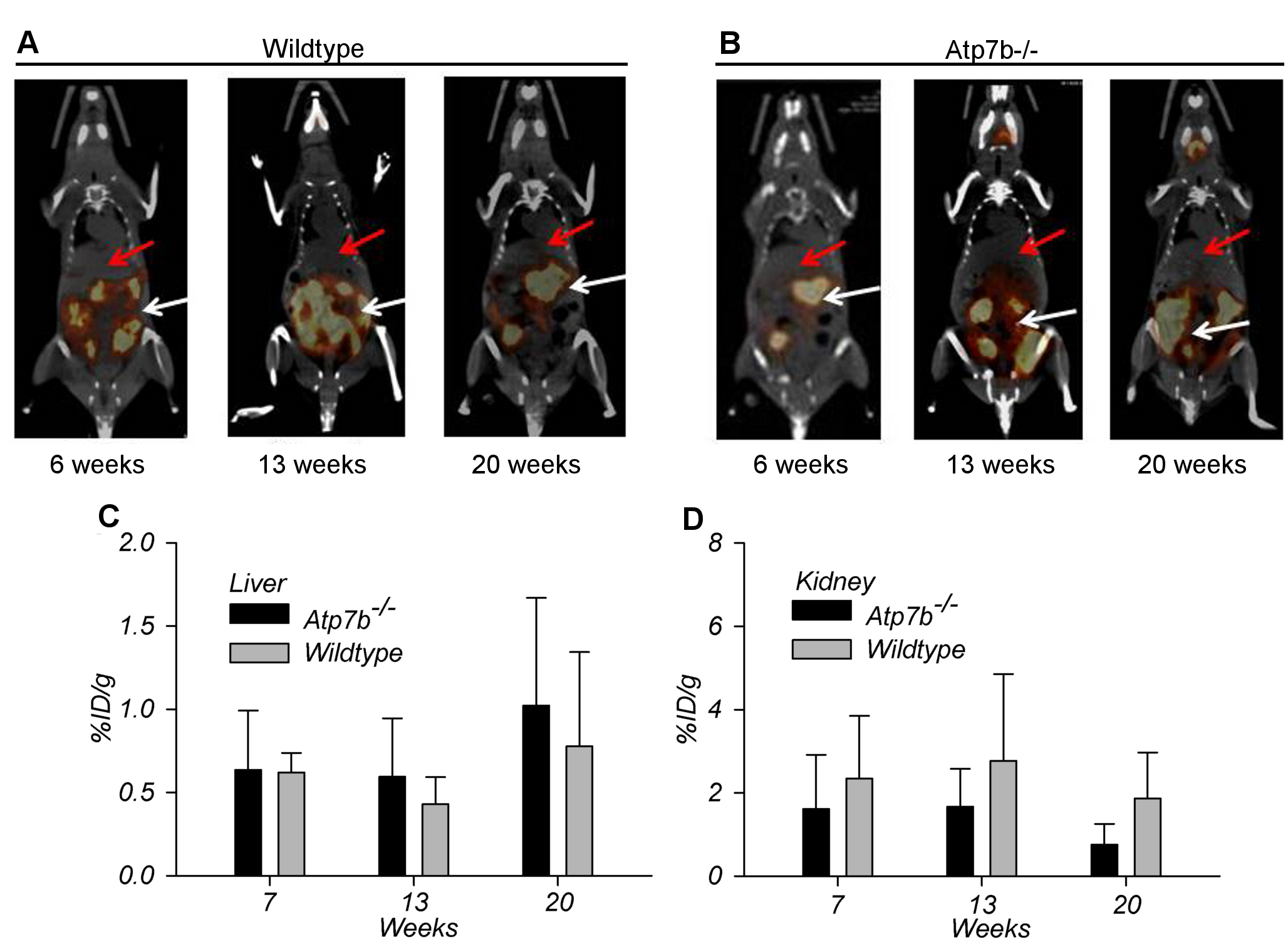

Supplement: Figure S2 — Related to Figure 3 . Representative PET-CT imaging and quantitative analysis of wildtype and Atp7b−/− mice at 2 hours post oral administration of 64Cu. Pet-CT images of (A) wildtype and (B) Atp7b−/− mice at indicated ages. Quantitative analysis of radioactivity of (C) liver and (D) kidneys of wildtype and Atp7b−/− mice at indicated ages (p = 0.27 for group effect). Data presented as mean ± SD. n = 5, per genotype. Red arrows indicate the position of the liver and white arrows indicate 64Cu radioactivity in the gastrointestinal tract. (TIF) [file pone.0038327.s002.tif]

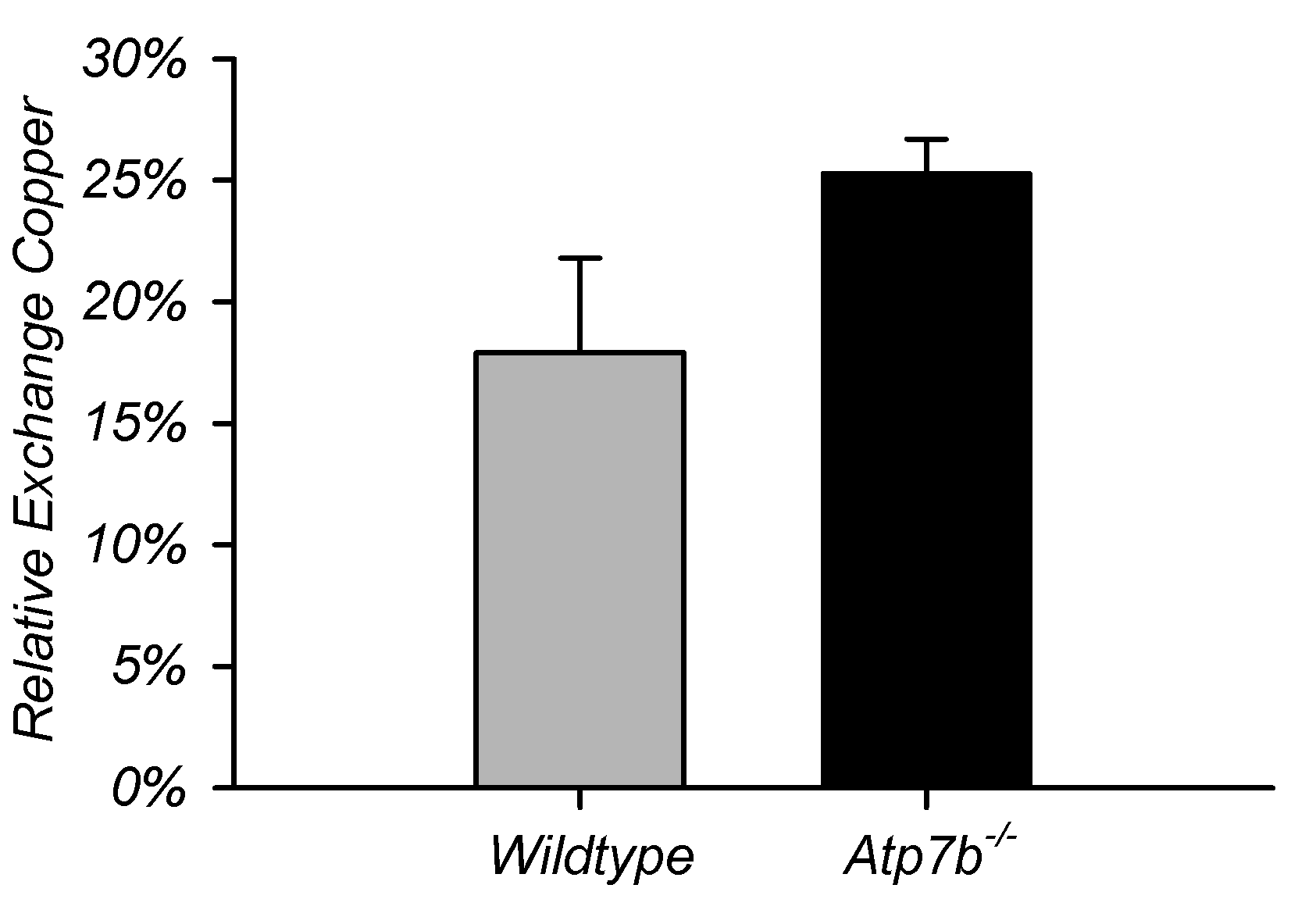

Supplement: Figure S3 — Related to Figure 7 : Exchangeable copper in the serum of wild-type and Atp7b−/− mice. Data presented as mean ± SD. n = 2 per genotype. See supporting information for experimental details (Information S1). (TIF) [file pone.0038327.s003.tif]
