# Supplementary material for: Urinary Copper Elevation in a Mouse Model of Wilson's Disease Is a Regulated Process to Specifically Decrease the Hepatic Copper Load
Source: PLoS One. 2012 Jun 22;7(6):e38327. doi: 10.1371/journal.pone.0038327 (PMC3390108; doi:10.1371/journal.pone.0038327)
Supplement: Table S1 — Related to Figure 4 : Correlation between changes in urine volume and amounts of urinary elements with disease progression. *Spearman ranked coefficients with significance p<0.05. n = 6. See supporting information for description of statistical analysis (Information S1). (DOCX) [file pone.0038327.s004.docx]

Table S1, related to Figure 4. Correlation between changes in urine volume and amounts of urinary elements with disease progression

| Element | Correlation coefficient (rho) | p-value* |
| --- | --- | --- |
| Na | 0.771 | 0.103 |
| Mg | 1.000 | 0.003 |
| P | 0.943 | 0.017 |
| K | 1.000 | 0.003 |
| Ca | 0.543 | 0.297 |
| Fe | 0.371 | 0.497 |
| Cu | 0.314 | 0.564 |
| Zn | 0.829 | 0.058 |
| Se | 1.000 | 0.003 |

*Spearman ranked coefficients with significance p<0.05. n=6.
