# Supplementary material for: Urinary Copper Elevation in a Mouse Model of Wilson's Disease Is a Regulated Process to Specifically Decrease the Hepatic Copper Load
Source: PLoS One. 2012 Jun 22;7(6):e38327. doi: 10.1371/journal.pone.0038327 (PMC3390108; doi:10.1371/journal.pone.0038327)
Supplement: Information S1 — Supplementary experimental procedures. (DOCX) [file pone.0038327.s005.docx]

**Supplementary Experimental Procedures**

**Statistical Analysis.**

Correlation coefficients between urine volume and element urinary amounts were calculated by Spearman ranked method analysis from 6 age groups. P-value is significant when p≤0.05. Box plot outliers were determined by multiplying the interquartile range by 1.5 and subtracting the obtained value from either the first quartile or third quartile, to determine the lower and upper limits; all measurements beyond these limits are considered outliers.

**Urinary protein measurements.**

Urine was collected as described in main text. The protein content of diluted urine (1:10) was measured by Bradford assay. The daily protein amounts were calculated by multiplying protein concentration by average daily urine volume for each animal. A combined total of 83 wildtype and Atp7b^-/-^ urine samples were assayed and grouped according to ages, 4-7 weeks, 8-14 weeks, 15-20 weeks, and greater than 20 weeks. N=3 to 15 samples per age-group and genotype.

**Relative exchangeable copper.**

Relative exchangeable copper (REC) was determined as described previously [[1](#_ENREF_1)]. Blood was collected from isoflurane anesthetized mice following superior vena cava incision and bleeding into thoracic cavity. Blood was immediately placed on ice and allowed to clot then centrifuged at 6k x g for 10 minutes to obtain serum. Serum from Atp7b-/- and control mice were separately mixed 1:1 with 3g/L EDTA and incubated at room temperature for exactly 1 hour. Mixture was placed into a YM-3 microcon and centrifuged at 4C for 90 minutes at 14k x g. Copper is measured in both filtrate and retentate by atomic absorption. Relatively exchangeable copper is determined by dividing filtrate copper by total serum copper.

**Supplementary References**

1. El Balkhi S, Trocello JM, Poupon J, Chappuis P, Massicot F, et al. (2011) Relative exchangeable copper: a new highly sensitive and highly specific biomarker for Wilson's disease diagnosis. Clin Chim Acta 412: 2254-2260.
